# Therapeutic isolation and expansion of human skeletal muscle-derived stem cells for the use of muscle-nerve-blood vessel reconstitution

**DOI:** 10.3389/fphys.2015.00165

**Published:** 2015-06-02

**Authors:** Tetsuro Tamaki, Yoshiyasu Uchiyama, Maki Hirata, Hiroyuki Hashimoto, Nobuyuki Nakajima, Kosuke Saito, Toshiro Terachi, Joji Mochida

**Affiliations:** ^1^Muscle Physiology and Cell Biology Unit, Tokai University School of MedicineIsehara, Japan; ^2^Department of Human Structure and Function, Tokai University School of MedicineIsehara, Japan; ^3^Department of Orthopedics, Tokai University School of MedicineIsehara, Japan; ^4^Department of Urology, Tokai University School of MedicineIsehara, Japan

**Keywords:** adult stem cell, Pax7, satellite cells, MyoD, p75, human nuclear antigen, muscle interstitium

## Abstract

Skeletal muscle makes up 40–50% of body mass, and is thus considered to be a good adult stem cell source for autologous therapy. Although, several stem/progenitor cells have been fractionated from mouse skeletal muscle showing a high potential for therapeutic use, it is unclear whether this is the case in human. Differentiation and therapeutic potential of human skeletal muscle-derived cells (Sk-Cs) was examined. Samples (5–10 g) were obtained from the abdominal and leg muscles of 36 patients (age, 17–79 years) undergoing prostate cancer treatment or leg amputation surgery. All patients gave informed consent. Sk-Cs were isolated using conditioned collagenase solution, and were then sorted as CD34^−^/CD45^−^/CD29^+^ (Sk-DN/29^+^) and CD34^+^/CD45^−^ (Sk-34) cells, in a similar manner as for the previous mouse Sk-Cs. Both cell fractions were appropriately expanded using conditioned culture medium for about 2 weeks. Differentiation potentials were then examined during cell culture and *in vivo* transplantation into the severely damaged muscles of athymic nude mice and rats. Interestingly, these two cell fractions could be divided into highly myogenic (Sk-DN/29^+^) and multipotent stem cell (Sk-34) fractions, in contrast to mouse Sk-Cs, which showed comparable capacities in both cells. At 6 weeks after the separate transplantation of both cell fractions, the former showed an active contribution to muscle fiber regeneration, but the latter showed vigorous engraftment to the interstitium associated with differentiation into Schwann cells, perineurial/endoneurial cells, and vascular endothelial cells and pericytes, which corresponded to previous observations with mouse SK-Cs. Importantly, mixed cultures of both cells resulted the reduction of tissue reconstitution capacities *in vivo*, whereas co-transplantation after separate expansion showed favorable results. Therefore, human Sk-Cs are potentially applicable to therapeutic autografts and show multiple differentiation potential *in vivo*.

## Introduction

Skeletal muscle organ/tissue comprises 40–50% of body mass, and it undergoes marked changes postnatally as a result of physical over-load or disuse, known as muscle hypertrophy and atrophy, respectively. Basically, skeletal muscle is composed of major four tissues as: (1) muscle fibers; (2) peripheral nerve networks; (3) blood vessel networks; and (4) connective tissue networks. Therefore, it is possible that the volume and/or cell number of these tissues are also changeable via whole muscle hypertrophy or atrophy. This phenomenon also suggests that skeletal muscle tissue must contain stem and/or progenitor cells that can support increases in the above tissues. Indeed, our previous studies have indicated that multipotent stem cell populations, which could synchronously reconstitute the muscle-nerve-blood vessel units following cellular differentiation into skeletal muscle cells, Schwann cells, perineurial/endoneurial cells, vascular endothelial/smooth muscle cells, pericytes and fibroblasts, are present in the mouse skeletal muscle (Tamaki et al., 2002, 2003, 2005, 2007a,b). Stem cells in skeletal muscle have been fractionated by various methods, including pre-plating culture series (Lee et al., 2000; Torrente et al., 2001; Qu-Petersen et al., 2002), repeated culture following the freeze-thaw technique (Williams et al., 1999; Young et al., 2001; Romero-Ramos et al., 2002) and fluorescence activated cell sorting (FACS) with cell surface makers (Tamaki et al., 2002) or with Hoechest dye (Gussoni et al., 1999; Jackson et al., 1999; Majka et al., 2003; Tamaki et al., 2003). However, because of these variations in isolation/fractionation methods, it is difficult to directly compare the origin, localization and differentiation potentials of these stem cells. Therefore, there are no standard methods for the isolation of practical stem cells from skeletal muscles.

Nevertheless, the ultimate aim of stem cell research is the safe clinical application of the transplantation therapy. For this purpose; in the present study, we fractionated human skeletal muscle-derived cells (Sk-Cs), taking advantage of the typical characteristics of our isolation methods; such as the soft cell isolation without mincing of muscles, and cell sorting in the freshly isolated state, following the previous series of mouse experiments (Tamaki et al., 2002, 2003, 2005, 2007a,b, 2008a,b, 2010). Appropriate cell markers for purification and culture conditions to obtain sufficient cell expansion were examined with regard to safety, rapidity and stability, as well as preservation of subsequent *in vivo* differentiation capacity.

Results indicated that the human Sk-Cs can be divided into three fractions, CD34^−^/CD45^−^/CD29^+^ (Sk-DN/29^+^), CD34^+^/CD45^−^/CD29^+^ (Sk-34/29^+^) and CD34^+^/CD45^−^/CD29^−^ (Sk-34/29^−^), similarly to mouse Sk-Cs. Interestingly, these cell fractions could also be divided into highly myogenic (Sk-DN/29^+^) and multipotent stem cell (Sk-34/29^+/−^) fractions, in contrast to mouse Sk-Cs. After separate transplantation of human Sk-DN/29^+^ and Sk-34/29^+/−^ cells into the damaged muscles of nude mice and rats, the former showed active contributions to muscle fiber regeneration, and the latter showed vigorous engraftment to the interstitium following differentiation into neural Schwann cells, perineurial/endoneurial cells, and vascular endothelial cells and pericytes. Therefore, the present preparation method for human Sk-Cs is potentially applicable to therapeutic autografts, thereby allowing efficient use of their multiple differentiation potentials *in vivo*, as was indicated by our previous mouse experiments.

## Materials and methods

### Human skeletal-muscle samples and cell isolation

Samples (5–10 g) were obtained from the abdominal, tibialis anterior, soleus, gastrocnemius, vastus-medialis, and vastus-lateralis muscles of 36 patients (age, 17–79 years) undergoing prostate cancer (*n* = 27) or leg amputation (*n* = 9) surgery. Study protocols were approved by our institutional ethics committee, and all patients gave consent after being informed of the study aims and procedures. Abdominal muscles were obtained from around the camera-port in laparoscopic surgery, and leg muscles were obtained from amputated, but retained non-damaged tissue portion. Muscle samples were wrapped in gauze moistened with cold (4°C) physiological saline immediately after removal, and were transferred to the laboratory for isolation of stem cells within 30 min.

Stem cells were isolated using a procedure corresponding to that previously described for mouse muscles (Tamaki et al., 2002, 2003). Briefly, muscle samples were weighed and washed several times with Dulbecco's modified essential medium (DMEM) with 1% penicillin/streptomycin, and were cut into several pieces (5–7 mm in thickness and width, and 40–50 mm in length). Muscles were never minced. Muscle pieces were treated with 0.1% collagenase type IA (Sigma-Aldrich, St. Louis, MO) in DMEM containing 7.5% fetal calf serum (FCS) with gentle agitation for 2 h at 37°C. Extracted cells were filtered through 70-μm, 40-μm and 20-μm nylon strainers in order to remove muscle fibers and other debris, and were then washed and resuspended in Iscove's modified Dulbecco's medium (IMDM) containing 10% FCS, yielding enzymatically extracted cells. Enzymatically extracted mixed cells were then, prepared for staining with cell surface antigens and sorting, or were stored in liquid nitrogen using cell preservative solution (Cell Banker; Juji-field, Tokyo, Japan) until use, after pre-freezing at −80°C using a bio freezing vessel (BICELL; Nihon Freezer Co., Ltd, Tokyo, Japan).

### Flow cytometry and sorting of enzymatically isolated cells

**Figure 1:**
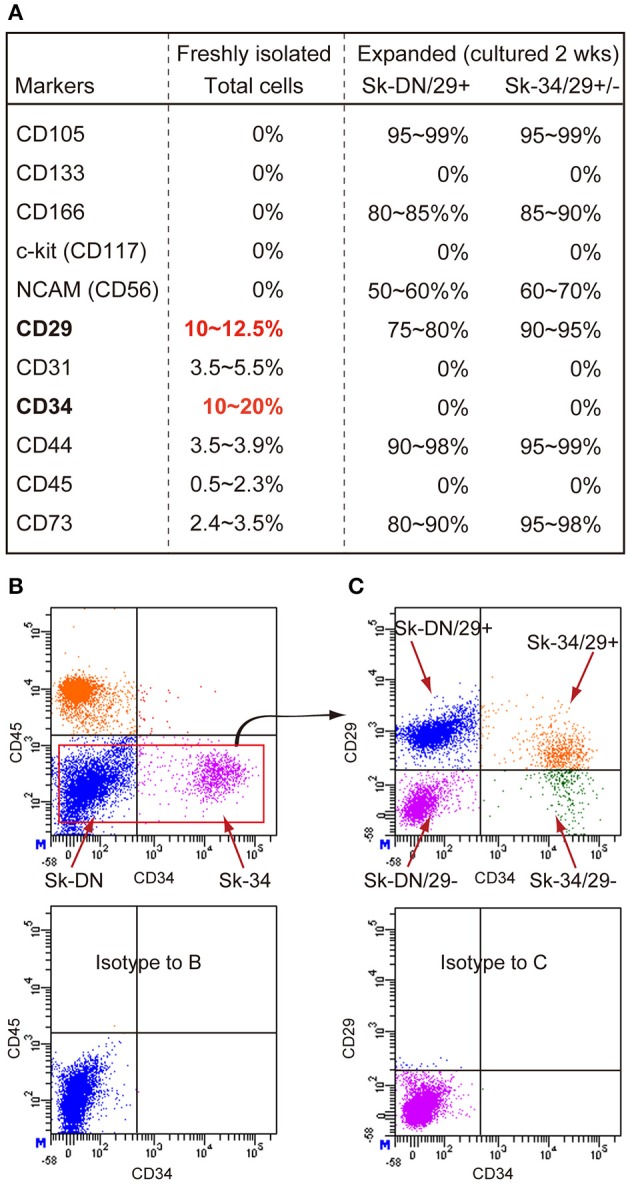
**Expression of CD markers in freshly isolated and expanded (passage 1) Sk-Cs. (A)** Freshly isolated total cells were positive for 6 out of 11 markers, and predominant markers were CD29 and CD34; thus, these were used for subsequent cell fractionation. However, expression of these markers markedly changed after culture of both Sk-DN/29^+^ and Sk-34/29^+/−^ cells, but importantly, the characteristics of both cells were similar after expansion. **(B)** Actual sorting for the exclusion of CD45^+^ cells, as hematopoietic cells. **(C)** Basic sorting pattern of 3 cell fractions, as Sk-DN/29^+^, Sk-34/29^+^ and Sk-34/29^−^, which was consistently used throughout the present study. Data were obtained from 21 subjects/32 samples (Male *n* = 19, age 21–79; Female *n* = 2, age 17–69, from abdominal muscle *n* = 16, leg muscles *n* = 5).

First, in order to characterize the enzymatically isolated cells and to determine the appropriate markers, FACS analysis was performed on freshly isolated human Sk-Cs using typical mesenchymal stem cell surface makers: CD29, CD31, CD44, CD56 (NCAM) and CD73 (purchased from BD Biosciences, Dan Jose, CA); CD105 and CD117 (c-kit) (from BioLegend, San Diego, CA); CD133 (from Miltenyi Biotec, Bergisch Gladbach, Germany); and CD166 (from Beckman Coulter, Brea, CA); as well as CD34 (RAM34; eBioscience, San Diego, CA) and CD45 (30-F11; BioLegend, San Diego, CA) antibodies. Cell analysis and sorting were carried out on a FACSAria (Becton Dickinson Japan, Tokyo, Japan). Note that the same cell characterization was also performed for expanded cells cultured under the conditions described blow. Consequently, based on the results for freshly isolated cells (see Figure 1A), we decided to sort human Sk-Cs using CD29, CD34 and CD45, and obtained the CD34^+^/45^−^/29^+^ (Sk-34/29^+^), CD34^+^/45^−^/29^−^ (Sk-34/29^−^), CD34^−^/45^−^/29^+^ (Sk-DN/29^+^), and CD34^−^/45^−^/29^−^ (Sk-DN/29^−^) fractions (see Figures 1B,C).

### Determination of proper culture condition and cytokines

In order to determine optimal culture conditions for cell expansion, we first selected 10 cytokines, as summarized in Supplemental Table 1, based on previous reports. Permutations and combinations of these cytokines were performed for the three cell fractions above using 24-well plates. IMDM was used as a basic culture medium. Evaluation was performed as follows; (1) 1000 cells/well were cultured to confluence; (2) cells were detached using 0.05% trypsin EDTA (Life Technologies, Tokyo, Japan), re-plated in 25-cm^2^ flasks, and cultured to confluence; and (3) cells were re-plated in 75-cm^2^ flasks and cultured to confluence. Expanded cells at each stage were photographed, and the time (days) to attain confluence were determined and compared.

On the other hand, some of these expanded cells were further cultured with repetitive passages (until 10 passages) and increasing culture terms. Finally, expanded cells were harvested and counted, and analyzed as follows; (1) FACS analysis (see above); (2) karyotype checking; (3) myotube formation test; (4) expression of specific mRNAs; and (5) transplantation analysis (see below).

### Karyotype checking

Karyotype abnormalities were examined using colchicine and Giemsa stain at various stages (passages and terms) of cell culture up to 10 passages. Cells were arrested in metaphase during cell-division with a solution of colchicine (0.01% for 6 h), harvested using 0.05% trypsin EDTA (Life Technologies), and pelleted by centrifugation at 1000 rpm for 5 min. Cells were re-suspended in hypotonic KCl solution (0.075 M) for 15 min at 37°C, and were fixed with Carnoy fixative. After repeated washing of cells, Giemsa staining was performed, and the stained cell pellet was dropped on a slide glass and dried at 56°C. Finally, 20 cells in metaphase were analyzed by G-banding in each selected sample.

### Myotube formation test

After determination of optimal culture conditions, we performed myotube formation testing in 3 cell fractions. Myotube formation was simply induced by reducing serum concentrations from 20 to 5% (Table 1) and by addition of HGF (20 ng/ml), as these conditions induced the most active myotube formation in human cells.

**Table 1:** **Optimal culture conditions for human Sk-34 and Sk-DN cells**.

	**Awaking medium**	**Proliferation medium (from passage 1)**	
	**Common use**	**Sk-34/29^+/−^fraction**	**Sk-DN/29+ fraction**
Basic contents	IMDM	IMDM	IMDM
	20%FBS	20%FBS	20%FBS
	0.1 mM 2-Mercaptethanol	0.1 mM 2-Mercaptethanol	0.1 mM 2-Mercaptethanol
	50 μg/ml gentamicin	50 μg/ml gentamicin	50 μg/ml gentamicin
Additional contents	0.4 ng/ml EGF	0.4 ng/ml EGF	0.4 ng/ml EGF
	12.5 ng/ml bFGF	12.5 ng/ml bFGF	12.5 ng/ml bFGF
	20 ng/ml IGF-1	20 ng/ml IGF-1	
	4 mg/ml Dexamethasone		

### RT-PCR analysis for expanded Sk-Cs

After optimal culture conditions were confirmed, RT-PCR was performed on expanded (passages 1–4) Sk-DN/29^+^, Sk-34/29^+^, and Sk-34/29^−^ cells in order to test for expression of specific markers of skeletal muscle, peripheral nerve and vascular cell lineages. Specific primers and analyzed materials are summarized in Supplemental Table 2. Cells were lysed and total RNA was purified using a QIAGEN RNeasy micro kit. First-strand cDNA synthesis was performed with an Invitrogen SuperScript III system using dT30-containing primer (see above), and specific PCR (35 cycles of 30 s at 94°C, 30 s at 60–65°C and 2 min at 72°C) was performed in a 15-μl volume containing Ex-Taq buffer, 0.8 U of ExTaq-HS-polymerase, 0.7 μM specific sense and antisense primers, 0.2 mM dNTPs and 0.5 μl of cDNA. Analysis was performed for 3–6 samples in each fraction and for each passage, and relative expression intensity was classified into 3 levels, based on the housekeeping-control gene (GAPDH). Obtained values were averaged.

### Transplantation into damaged tibialis anterior (TA) muscle

In order to determine *in vivo* differentiation potential, optimally cultured-expanded (2 passages) human Sk-DN/29^+^ and Sk-34/29^+/−^ cells were transplanted into the severely damaged TA muscle model, which was removed about 30–40% of muscle fragments with nerve and blood vessel branches from the region around the motor point (Tamaki et al., 2005). For *in vivo* transplantation analysis, sufficient numbers of Sk-34/29^−^ cells could not be obtained through the same process as the other two cell fractions. In addition, it was confirmed by RT-PCR analysis that the characteristics of Sk-34/29^−^ cells did not affect the total characteristics of Sk-34/29^+^ cells. Thus, we combined the Sk-34/29^+^ and /29^−^ cell fractions, and cultured/expanded them together, and then performed the transplantation study. Cells were transplanted into traumatic muscle damage with large deficits in muscle-nerve-blood vessel units in the athymic nude mouse (female, BALB/cA Jcl-*nu/nu*; CLEA Japan, Tokyo, Japan, age 5–6 wk, *n* = 14) and/or rat (male and female, F344/NJcl-mu/mu; CLEA, Tokyo, Japan, age 8–12 wk, *n* = 4) TA muscle. Surgery and cell transplantation were performed under inhalation anesthesia (Isoflurane; Abbott, Tokyo, Japan). The left TA muscle of nude mice was exposed by skin incision, and its fascia was minimally cut. Using forceps, muscle fibers with nerve and blood vessels were manually removed (17.1 ± 0.9 mg) from the region surrounding the motor point of the TA muscle. The large incision were sutured, and then cells (5.2 ± 1.5 × 10^5^) suspended in 2~3 μl of DMEM were injected slowly into the damaged muscle portion using a fine tip glass micropipette, in order to avoid diffusion of transplanted cells. Then, a transparent sterile/analgesic plastic dressing (Nobecutan spray; Yoshitomi Chemical, Japan) was sprayed over the wound. The right TA muscle was preserved as a control. At 6–8 weeks after transplantation, recipient mice were given an overdose of pentobarbital (60 mg/kg, i.p.), and were perfused with warm 0.01 M phosphate-buffered saline (PBS) through the left ventricle, followed by fixation with 4% paraformaldehyde/0.1 M phosphate buffer (4% PFA/PB). TA muscles were then removed and re-fixed overnight in 4% PFA/PB, washed with graded sucrose 0–25%/0.01 M PBS series, and were quick frozen in isopentane pre-cooled with liquid nitrogen, followed by storage at −80°C until use. This *in vivo* cell engraftment/differentiation analysis were performed using the samples of 16 subjects (male: *n* = 14, age 17–79; female: *n* = 2, age 17; 8 abdominal and 8 leg muscles) with 52 times transplantations. All experimental procedures were approved by the Tokai University School of Medicine Committee on Animal Care and Use (No. 142035, 142034). All experiments were performed in the Tokai University School of Medicine and Hospital.

### Immunocytochemistry, immunohistochemistry, and immunoelectron microscopy

Cell differentiation potential *in vitro* was examined by immunocytochemistry. For the staining of cultured cells, each culture well was fixed with 4% PFA/PB for 10 min. After washing with 0.01 M PBS, cells were stained with anti-Pax7 (mouse monoclonal, 1:50 overnight at 4°C; Developmental Studies Hybridoma Bank, University of Iowa, Iowa, IA) or MyoD (mouse monoclonal, 1:50, overnight at 4°C, 5.8A; DAKO, Carpinteria, CA) to detect the myogenic cells and putative muscle satellite cells, respectively. Similarly, cultured cells were suspended in trypsin-EDTA and prepared for cytospin analysis.

Cell differentiation potential *in vivo* was examined by immunohistochemistry and immunoelectron microscopy. For staining of histological sections, several 7−μm cross-sections of operated TA muscles were obtained. Engrafted human cells were detected by anti-human nuclear antigen (HNA, 1:100, 4°C overnight; clone 235-1, Cy3 conjugate, Millipore, Temecula, CA). Blood vessels were detected using mouse monoclonal αSMA (Cy3-conjugated directly; 1:1500, for 1 h at room temperature; Sigma, Saint Louis, MO), rat anti-mouse CD31 (1:500, 4°C overnight; BD Biosciences, San Jose, CA), and rabbit polyclonal anti-human CD31 (1:200, for 2 h at room temperature, Abcam, Cambridge, UK). Pericytes were detected by the combination use of rabbit polyclonal anti-NG2 (1:100, 1 h at room temperature; Millipore) and anti/human CD31. Immature and/or newly differentiated Schwann cells were detected using rabbit anti-p75 (1:400, 4°C overnight; CST, Boston, MA) polyclonal antibody, in relation to the myelin formations detected by rabbit polyclonal anti-myelin basic protein (MBP; 1:200, for 2 h at room temperature; Millipore, Billerica, MA). Muscle fibers were detected with rabbit polyclonal anti-skeletal muscle actin (1:300, for 1 h at room temperature; Abcam, Cambridge, UK). Rabbit polyclonal anti-laminin (Pan-laminin, 1:1500 for 2 h at room temperature, LSL, Tokyo, Japan) and/or rat monoclonal anti mouse laminin (β-2 chain, 1:2000 for 2 h at room temperature, Chemicon, Temecula, CA) antibodies were used to distinguish muscle fibers and interstitial spaces. Reactions were visualized using Alexa Fluor-488- and 594- conjugated goat anti-rabbit and anti-rat antibodies (1:500, for 2 h at room temperature; Molecular Probes, Eugene, OR). Nuclei were counter-stained with DAPI (4′,6-diamino-2-phenylindole).

For immunoelectron microscopy, sections were stained using anti-HNA (1:50, 4°C overnight; clone 235-1, biotin conjugate; Millipore) and followed by HRP-conjugated streptavidin second antibody (1:200, for 1 h at room temperature; DAKO) in order to label engrafted human cells. Reactions were visualized with DAB (3, 3′-Diaminobenzidine) after fixation in 1% glutaraldehyde/0.1 M phosphate buffer. Visualized sections were then fixed in 1% osmium tetroxide/0.05 M phosphate buffer, and were prepared for electron microscopic analysis. Cell differentiation into Schwann cells, perineurial/endoneurial cells, vascular endothelial cells and pericytes, and fibroblasts were mainly observed. Immunoelectron microscopy was performed as described previously (Tamaki et al., 2005, 2007a, 2008a,b, 2010).

### Quantitative analysis

Cells specifically determined by immunocytochemistry on cultured and/or cytospin preparation were counted using Photoshop (Adobe Systems Inc., San Jose, CA) or Stereo Investigator (MBF Bioscience, Williston, VT) software. Immunohistochemically positive/negative cells were photographed by the unit area to wholly cover all distributing cells (6–12 area), and were averaged and expressed as percentages (positive/total cells).

## Results

### Cell characterization by CD markers for the freshly isolated and after expansion culture state

Freshly isolated human Sk-CDs were characterized using 11 candidate CD makers, and they were typically negative for CD105, CD133, CD166, c-kit, and NCAM (CD56) (Figure 1A). Less than 5% positivity was observed for CD31, CD44, CD45 and CD73, and over 10% positivity was observed for CD29 and CD34. Therefore, we decided to sort human Sk-Cs using CD29 and CD34, in addition to CD45, due to its elimination role for hematopoietic cells. The basic sorting pattern for freshly isolated human Sk-Cs is shown in Figures 1B,C. Human cells showed a similar pattern as mouse cells under sorting by CD45 and CD34 (Figure 1A), which was reported previously (Tamaki et al., 2002, 2003, 2005, 2007a,b, 2008b). No CD34^+^/CD45^+^ cells were seen among human Sk-Cs, and this is a difference from mouse Sk-Cs (Figure 1B). Cells in the Sk-DN and Sk-34 fractions were further divided into 4 fractions; CD34^+^/45^−^/29^+^ (Sk-34/29^+^), CD34^+^/45^−^/29^−^ (Sk-34/29^−^), CD34^−^/45^−^/29^+^ (Sk-DN/29^+^), and CD34^−^/45^−^/29^−^ (Sk-DN/29^−^) fractions (Figure 1C). We have previously sorted freshly isolated cells from murine skeletal muscle by CD34 and CD45, and simply obtained Sk-34 and Sk-DN cells (Tamaki et al., 2002, 2003, 2005, 2007a,b, 2008b). In this method, however, the Sk-DN fraction tended to include debris and unnecessary non-stem cells. This contaminating debris must be avoided in human therapy. As expected, the Sk-DN/29− fraction included debris and non-proliferative cells; thus, we eliminated this fraction from further experiments.

Figure 1A also shows a comparison of marker expression between freshly isolated and 2-wk (passage 1) expanded Sk-DN/29^+^ and Sk-34/29^+/−^ cells. These expanded cells were subjected to further *in vitro* and *in vivo* analysis. Note that the characteristics of these 2 cell populations after expansion culture were similar. Both cell populations consistently expressed a high percentage of CD105, CD166, NCAM (CD56), CD29, CD44 and CD73, and the remaining markers showed 0% positivity. Although these populations showed the same cell surface markers, there were differences in the *in vitro* and *in vivo* cell differentiation capacities (see below).

### Determination of optimal culture conditions

Through a series of experiments, we determined the optimal awaking and proliferation media for the Sk-34 and Sk-DN cell fractions, as shown in Table 1. Basically, activation of human cells occurred more slowly when compared with mouse cells (4–7 days). Therefore, the awaking medium is important, and was the same for both the Sk-DN and Sk-34 cell fractions. However, the proliferation medium differed, and these optimal conditions were selected to give higher proliferation maintaining cell immaturity, as progressive differentiation during culture reduces the potential for tissue engraftment and reconstitution. Consequently, 300~ 450-fold increases in the number of cells were achieved during the 14–16-day culture period, through 1–3 passages, with 9–12 cell doublings. We consistently used these culture conditions in further *in vitro* and *in vivo* experiments.

With regard to karyotyping through long-term cell culture (around 10 passages), we detected abnormalities in 4 cases; 1 cases in Sk-DN/29^+^ cells (deletion of short arm chromosome 17), and 3 cases in unsorted bulk cell cultures (trisomy of chromosome 8, translocation of chromosome 10 and 11, translocation of chromosome 18 and 20). There were no abnormalities found in the Sk-34 cell fractions. The common factors in these 4 cases were continuous use of dexamethasone in the proliferating culture medium over 5 passages (mainly appeared in 8–9 passages) and over 40 days of the total culture term. However, use of dexamethasone in the awaking medium in the first week of culture is effective for both Sk-DN and Sk-34 cell expansion and safety. Therefore, we eliminated dexamethasone from the proliferating medium (see Table 1). In addition, unsorted bulk cell culture was also a risk factor; thus, it should be avoided. Cell sorting before culture is therefore important. Consequently, the awaking and proliferating culture conditions were modified to avoid all of these risk factors.

Furthermore, we eliminated HGF because it accelerated myotube formation in both Sk-DN and Sk-34 cells. This was considered to be unsuitable for maintaining an immature state, which is necessary for therapeutic use.

### Behaviors of Sk-DN/29^+^, Sk-34/29^+^, and Sk-34/29^−^ cells *in vitro*

**Figure 2:**
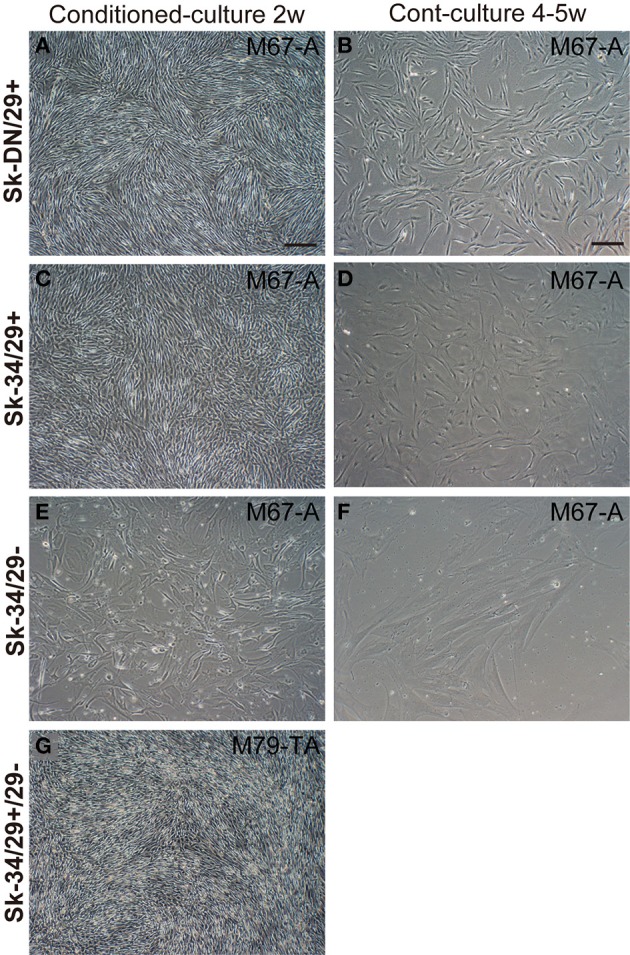
**Typical cellular behaviors in culture for three cell fractions. (A,B)** Sk-DN/29^+^, **(C,D)** Sk-34/29^+^, **(E,F)** Sk-34/29^−^, and **(G)** Sk-34 (29^+/−^). Cell behaviors were apparently different in the 4 cell fractions. The left column shows the results after 2 weeks of expansion culture in conditioned medium. The right column shows control medium (IMDM/20%FCS with antibiotics only) without any additional cytokines. Favorable cell proliferation was apparent in conditioned medium in each cell fraction, as compared to control. However, Sk-34/29^−^ cells showed significantly lower proliferation **(E)**, but when two Sk-34 cells (29^+/−^) were prepared for combined culture, improved confluence of cells was obtained **(G)**. Behaviors of Sk-DN/29^−^ cells were also presented in Supplemental Figure 1. Bars = 100 μm. In this analysis, over 1250 well plates from 22 subjects/38 samples (Male *n* = 20, age 21–79; Female *n* = 2, age 17–69, from abdominal muscle *n* = 16, leg muscles *n* = 6) were used.

Typical cellular behaviors in the three cell fractions are shown in Figure 2. Comparisons were performed in the 25-cm^2^ flask (see Methods). Cell behaviors differed in the Sk-DN/29^+^ (Figures 2A,B), Sk-34/29^+^ (Figures 2C,D), Sk-34/29^−^ (Figures 2E,F) and Sk-34 (29^+/−^, Figure 2G) cell fractions. The former two cell fractions showed full confluence after 2 weeks of conditioned culture with 1 passage (Figures 2A,C), whereas Sk-34/29^−^ cells showed 50–60% cell density (Figure 2E). When these cells were cultured in normal control medium (with 20%FCS only), less than 50% cell density was obtained with both Sk-DN/29^+^ (Figure 2B) and Sk-34/29^+^ (Figure 2D) cells, even after 4–5 weeks of culture, which was 2-times longer than with conditioned medium. Furthermore, Sk-34/29^−^ cells did not grow well in normal control media (Figure 2F). This difference demonstrates the efficacy of the present conditioned culture method. Owing to the lower increasing ratio for Sk-34/29^−^ cells, we performed combined culture of Sk-34/29^+^ and /29^−^ cell fractions using the same procedure (Figure 2G), and obtained better confluence than with solo culture of Sk-34/29^+^ cells (Figures 2C,G). Thus, combined expansion of total Sk-34 cells (unsorted by CD29) was more useful for further study and therapeutic use. Interestingly, co-expansion culture of Sk-DN/29^+^ and Sk-34 cells showed accelerated differentiation/commitment, and greater cell numbers were obtained (data not shown). Thus, it was considered to be unsuitable for further therapeutic cell expansion.

**Figure 3:**
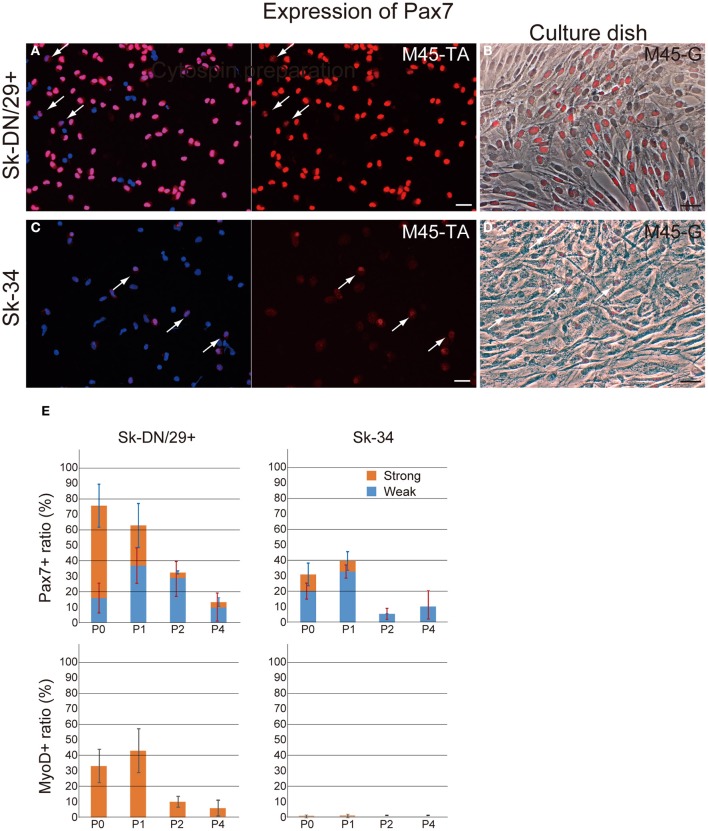
**Distribution of Pax7- and MyoD-positive cells in the Sk-DN/29^+^ and Sk-34 fractions**. Typical expression of Pax7 in Sk-DN/29^+^ **(A,B)** and Sk-34 **(C,D)** cells at passage 1. **(A,C)** Cytospin preparation. **(B,D)** Culture dish preparation. A large number of Pax7^+^ cells were observed among Sk-DN/29^+^ cells, but few were seen among Sk-34. Note that cells positive for Pax7 typically contained several weak reactions (arrows in **A** and **C**). This trend did not change in either cytospin or culture dish preparations. **(E)** Serial changes in the number of Pax7^+^ and MyoD^+^ cells from PO to P4, expressed as percentages of total cells are shown. Strong myogenic potential was detectable in Sk-DN/29^+^ cells, but was weak in Sk-34. Bars = 50 μm. Data were obtained from 11 samples (M40-VM, VL; M45-TA, G; M57-A; M58-A; M59-A; M61-A; F17-TA, S, G).

Subsequently, the distribution of putative satellite cells (Pax7-positive cells) and myogenically committed cells (MyoD-positive cells) were determined by the combined use of immunocytochemical analysis for cytospin, and cultured Sk-DN/29^+^ and Sk-34 cells preparations at passages 0–4 (Figure 3). A large number of Pax7^+^ cells were observed among Sk-DN/29^+^ cells (Figures 3A,B), but few were present among Sk-34 cells (Figures 3C,D), both on cytospin (Figures 3A,C) and cell culture (Figures 3B,D). Interestingly, Pax7 expression could be clearly divided into strong and weak reactions. Sk-DN/29^+^ cells also showed stronger Pax7 reactions, with few weak reactions (arrows in Figure 3A). In contrast, the total number of Pax7^+^ cells in the Sk-34 fraction was apparently less than that in Sk-DN/29^+^ cells (Figures 3A,C), and most showed weak reactions (arrows in Figure 3C). The same trend was confirmed under culture conditions, as there were no strong reactions among the Sk-34 cells (arrows in Figure 3D, as compared to Figure 3B). The number of Pax7- and MyoD-positive cells in both cell fractions were counted following passages 0–4 (Figure 3E). Pax7^+^ cells were evaluated as either strong or weak reactions. Among Sk-DN/29^+^ cells, over 75% of total Pax7^+^ cells were observed in P0, and gradually decreased with each passage (P1 = 62%, P2 = 32%, and P4 = 15%) and this trend did not change when it was evaluated by strong reactions only. In addition, active myogenic commitment accounting for 30–40% MyoD^+^ cells was observed at P0 and P1; however, this markedly decreased after P2. In contrast, in Sk-34 cells, total Pax7^+^ cells were counted around 30–40% during P0–P1, and clearly decreased after P2. Similarly, strong reactions for Pax7 disappeared after P2, while no myogenic commitment (detection of MyoD^+^ cells) was observed through Sk-34 cell expansion culture. Thus, myogenic commitment of Sk-34 cells appears to be rare *in vitro*.

**Figure 4:**
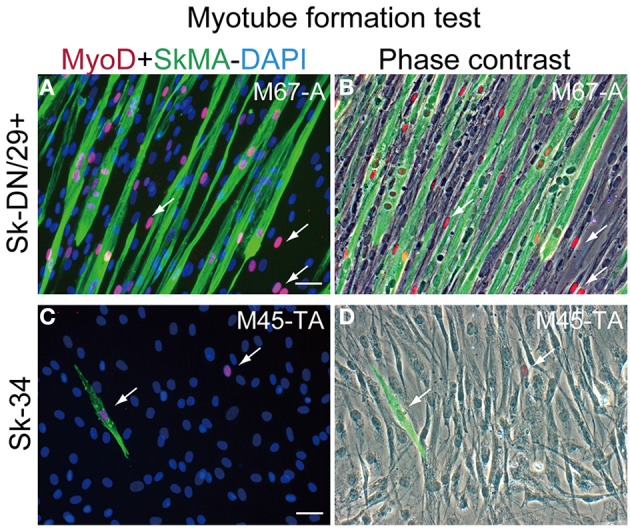
**Myotube formation test for Sk-DN/29^+^ and Sk-34 cells. (A,B)** Sk-DN/29^+^. **(C,D)** Sk-34 cells. There were numerous multinucleated myotubes formed in Sk-DN/29^+^ cells, but very few in Sk-34 cells. Myogenic potential was strong in Sk-DN/29^+^ cells, but was quite weak in Sk-34 cells. Single MyoD-positive cells (arrows) were also observed. Bars = 50 μm. Data were obtained from 11 samples (M67-A, VL; M45-TA, G; M57-A; M58-A; M59-A; M61-A; F17-TA, S, G).

Myotube formation test was also performed for Sk-DN/29^+^ and Sk-34 cells (Figure 4). Sk-DN/29^+^ cells showed active formation of myotubes associated with the expression of MyoD and SkMA (Figure 4A). Solo MyoD^+^ cells also showed cellular elongation (arrows in Figures 4A,B). These responses demonstrate the high myogenic potential of Sk-DN/29^+^ cells *in vitro*. In contrast, Sk-34 cells showed no multinucleated myotube formation, and few MyoD^+^ cells were observed; however, there were SkMA^+/−^ cells present (arrows in Figures 4C,D), suggesting lower myogenic potential. This myotube formation capacity gradually decreased following cell passage, even in Sk-DN/29^+^ cells (data not shown). Therefore, it is likely that 2–3 passages is the limit for the present cell expansion method for myogenic therapeutic use.

### RT-PCR analysis

**Figure 5:**
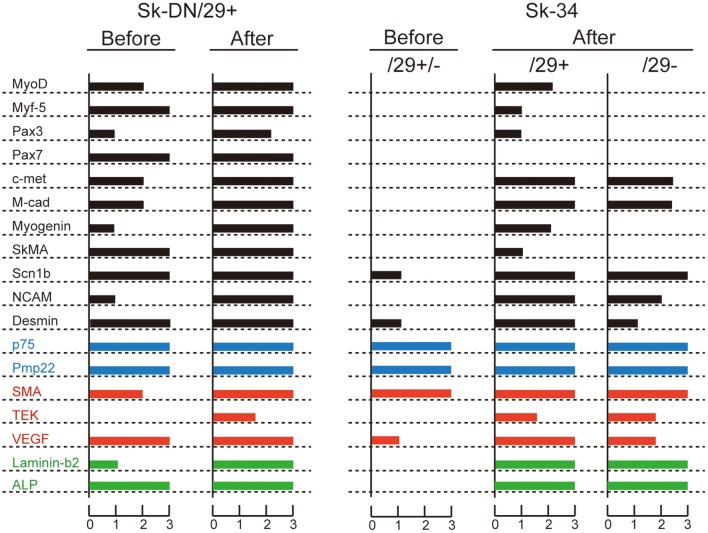
**RT-PCR analysis of Sk-DN/29^+^ and Sk-34 cells before and after expansion culture**. Bars in black color shows myogenic markers, blue shows peripheral nerve, and red shows vascular markers. Green shows common markers in the three lineages. Expression intensity was normalized based on the housekeeping-control gene (GAPDH), and values were averaged. These data supported the results of immunocytochemical analysis and myotube formation test. In addition, it was clear that Sk-DN/29^+^ cells comprised already committed myogenic cells in a freshly isolated state (see before), but Sk-34 cells did not include myogenic cells. Data were obtained from 21 subjects/36 samples (Male *n* = 20, age 21–79; Female *n* = 1, age 17, from abdominal muscle *n* = 16, leg muscles *n* = 5).

In order to support the immunocytochemical data, we performed RT-PCR analysis in relation to skeletal myogenic, peripheral nerve and vascular lineage makers for the 3 cell fractions before and after culture (Figure 5). In the freshly sorted state (before culture), Sk-DN/29^+^ cells expressed skeletal myogenic marker mRNAs, whereas Sk-34/29^+/−^ cells showed non-skeletal myogenic characteristics. Please note that the Sk-34 fraction before culture was tested as total Sk-34 cells (CD29^+/−^). After culture (P1 stage), however, differences were detected in the expression of skeletal myogenic markers (black bars), and other peripheral nerve (blue bars) and vascular (red bars) makers were similar in the 3 groups. Expression of c-met, M-cad, Scn1b and NCAM were similar in the 3 groups; however, there was no expression of MyoD, Myf-5, Pax3, Pax7, Myogenin and SkMA in the Sk-34/29^−^ cells. However, this expression was strong in Sk-DN, and weak in the Sk-34/29^+^ cells. Lack of Pax7 expression was common in both the Sk-34/29^+^ and /29^−^ fractions. These results correspond to the results for immunocytochemistry and myotube formation (Figures 3, 4). Therefore, Sk-DN cells showed strong myogenic potential, while Sk-34/29^+^ showed weak potential, and Sk-34/29^−^ cells showed none. These expression patterns after culture did not largely change through 5 passages. Therefore, taken together with the above immunocytochemical data, mRNA expression levels do not appear to be affected by cell passage, whereas protein expression levels are markedly reduced with each passage.

The characteristics of Sk-34/29^−^ cells appeared to be consistent with those of Sk-34/29^+^ cells with regard to protein and mRNA levels, and they were thought to compatible during cell expansion culture. Therefore, combined culture of total Sk-34 cells was considered to be suitable, because of the higher efficiency of cell expansion (see Figure 2) for therapeutic purposes.

### *In vivo* differentiation capacity after transplantation into severely damaged skeletal muscle

**Figure 6:**
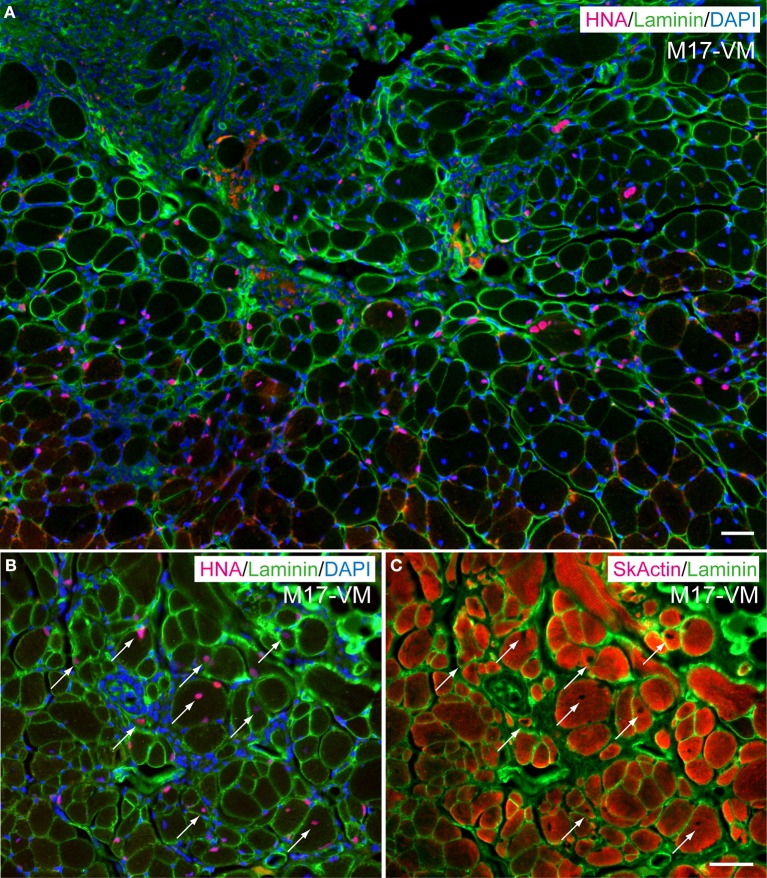
**Typical engraftment of Sk-DN/29^+^ cells in damaged muscle at 6 weeks after transplantation. (A)** Wide view of the transplanted portion. Pink = HNA (human nuclear antigen), green = laminin (basal lamina staining) and blue (nuclear staining by DAPI). Most HNA^+^ nuclei were located in the basal lamina of muscle fibers. Panels **(B,C)** show the same areas stained with HNA + laminin + DAPI **(B)**, and SkActin (skeletal muscle actin) + laminin **(C)** taken at higher magnification. HNA^+^ nuclei were clearly located in the muscle fibers (arrows). M17-VM = Male 17 year-old, vastus lateralis. Bars = 50 μm.

**Figure 7:**
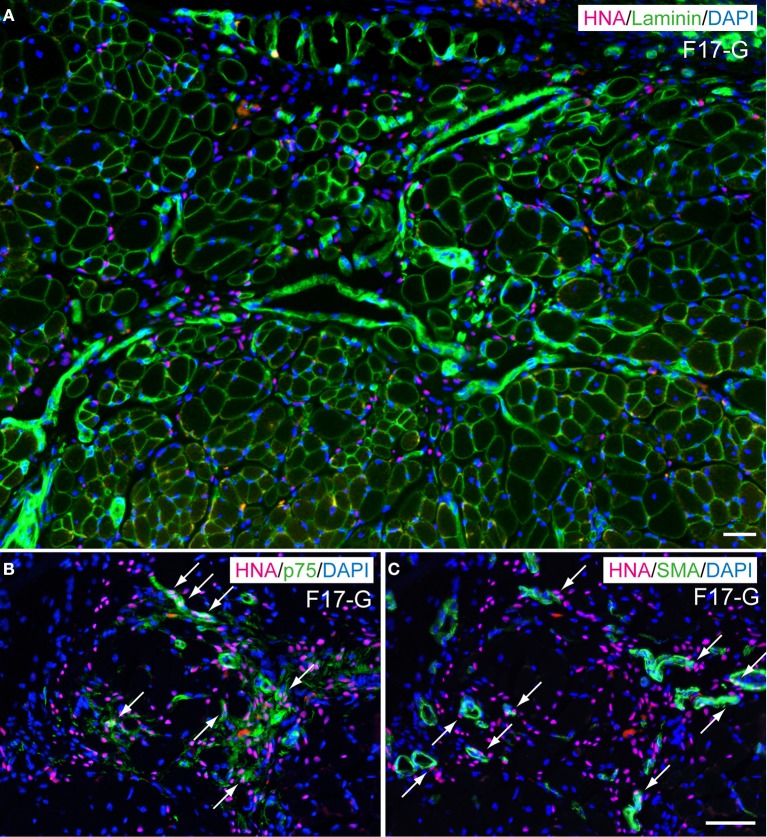
**Typical engraftment of Sk-34 cells in damaged muscle at 6 weeks after transplantation. (A)** Wide view of transplanted portion. Pink = HNA (human nuclear antigen), green = laminin (basal lamina staining), and blue (nuclear staining by DAPI). Most HNA^+^ nuclei were located in the interstitial spaces of muscle (outside of muscle fiber basal lamina). **(B,C)** show the same areas stained with HNA + p75 (green, Schwan cell marker) + DAPI **(B)**, and HNA + SMA (green, smooth muscle actin, vascular smooth muscle marker) + DAPI **(C)** taken at higher magnification. Co-staining with HNA and p75 was evident, showing Schwann cell differentiation of engrafted human cells (arrows in **B**). Similar co-staining was also observed in (**C**, arrows), thus suggesting differentiation of engrafted cells into vascular smooth muscle cells. F17-G = Female 17 year-old, gastrocnemius. Bars = 50 μm.

In order to determine *in vivo* cell differentiation capacity, both Sk-DN/29^+^ and total Sk-34 cells were transplanted into severely damaged TA muscle (Figures 6, 7). At 4–6 weeks after transplantation, a large number of HNA^+^ myofibers nuclei were seen in Sk-DN/29^+^ cell-transplanted muscles, and there were few HNA^+^ nuclei in the interstitium (Figure 6A). HNA^+^ nuclei were located inside the fiber basal lamina filled with SkMA (Figures 6B,C). In contrast, after Sk-34 cell transplantation, most HNA^+^ nuclei were distributed in the interstitium, and there were very few inside the muscle fibers (Figure 7A). Interstitial HNA^+^ nuclei were mainly distributed around the p75^+^ Schwann cells (Figure 7B) and/or SMA^+^ blood vessels (Figure 7C). Some of the p75^+^ Schwann cells included HNA^+^ nuclei, representing donor human cell-derived Schwann cell formations (arrows in Figure 7B). Similarly, some HNA^+^ nuclei were located near SMA^+^ blood vessels (arrows in Figure 7C).

**Figure 8:**
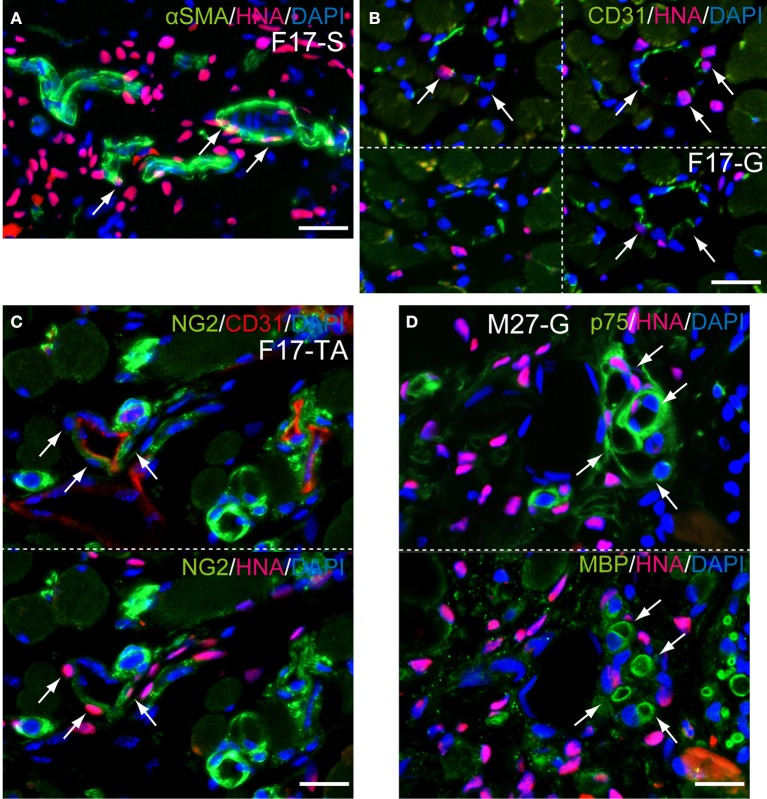
**Detection of cell differentiations by higher magnification immunofluorescences at 6 weeks after Sk-34 cell transplantation**. (**A**, arrows) SMA^+^/HNA^+^ cells located around blood vessel showing differentiation of engrafted human cells into vascular smooth muscle cells. (**B**, arrows) Anti-human CD31^+^/HNA^+^ cells were also observed around the blood vessel, showing differentiation into vascular endothelial cells. (**C**, arrows) NG2^+^/HNA^+^ cells located just around the mouse CD31^+^ blood vessel, showing differentiation into pericytes. (**D**, arrows) p75^+^/HNA^+^ cells were also located close to myelin (MBP^+^), showing differentiation into Schwann cells. F17-S, G, TA = Female 17 year-old, soleus, gastrocnemius, tibiaris aterior. Bars = 20 μm.

**Figure 9:**
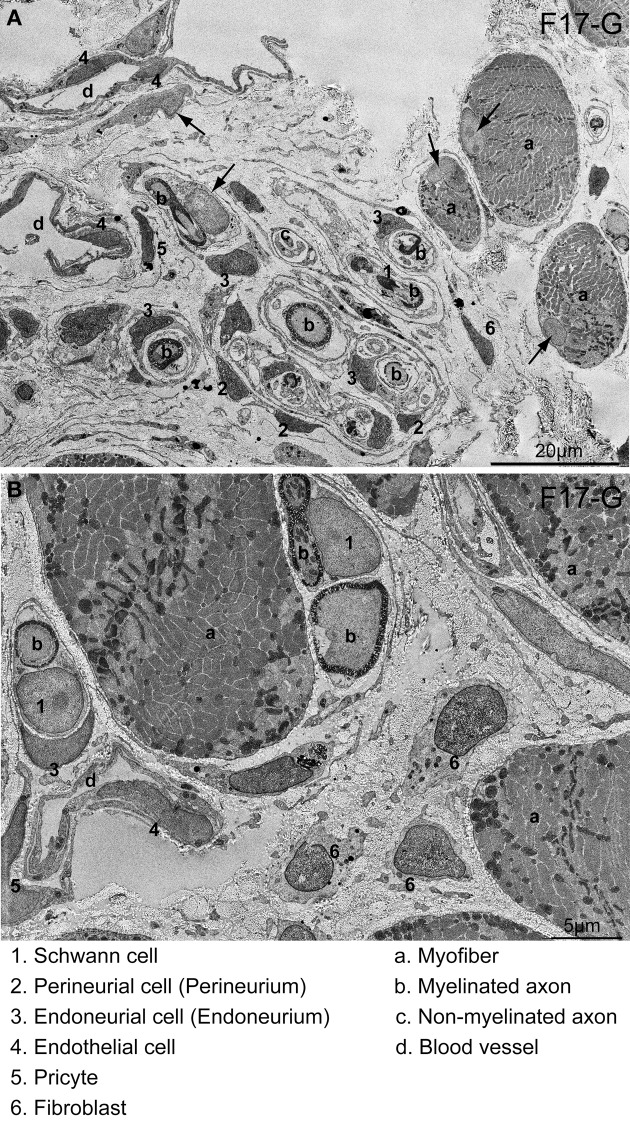
**Detection of cells having HNA^+^ nuclei following 6 weeks of Sk-34 cell transplantation by immunoelectron microscopy. (A)** Relatively wide interstitium.**(B)** Relatively narrow interstitium. HNA^+^ reactions were visualized with DAB; thus, they appeared as black dots. Numbers and letters inserted in the panels correspond to cells and/or tissues listed on the bottom of the photos. Differentiation into 1–6 cell lineages was confirmed. Arrows in **(A)** show HNA-negative nuclei, which is free of black dots. Details of the determination of HNA^+^ cells in the immunoelectron microscopy are presented in Supplemental Figure 2. F17-G = Female 17 year-old, gastrocnemius. Bars in **A** = 20 μm, and in **B** = 5 μm.

Detailed differentiation of interstitial cells having HNA^+^ nuclei following Sk-34 cell transplantation was also confirmed by the higher magnification immunofluorescence and immunoelectron microscopy (Figures 8, 9). The SMA^+^ cells including HNA^+^ nuclei were evident (Figure 8A, arrows), and human CD31^+^ reactions were also observed closely around the HNA^+^ nuclei (Figure 8B, arrows). In addition, NG2^+^ cells were also HNA^+^ just around the mouse CD31^+^ reactions (Figure 8C, arrows). These results indicated that engrafted Sk-34 cells differentiated into vascular smooth muscle cells, endothelial cells and pericytes, and contributed to the formation of blood vessels. Similarly, HNA^+^/p75^+^ cells close to MBP^+^ reactions were evident (Figure 8D, arrows) showing the differentiation into Schwann cells, and contribution to nerve regeneration.

In the immunoelectron microscopy, HNA^+^ reactions were visualized by DAB; thus, they appeared as black dots in immunoelectron micrographs, thus, it made darker nuclei (compare between numbered and arrowed nuclei in Figure 9A). Distributions of HNA^+^ cells were quite similar to immunofluorescence. Donor human Sk-34 cells differentiated into Schwann cells (1), perineurial/endoneurial cells with perineurium/endoneurium (2, 3), and vascular endothelial cells (4), pericytes (5) and fibroblasts (6), and contributed to peripheral nerve and blood vessel regeneration (Figures 9A,B).

We also performed the same transplantation of unsorted and expanded cells, which supposed to be included Sk-DN and Sk-34 cells, but they exhibited a reduced engraftment ratio and tissue reconstitution capacity, probably due to their accelerated cell differentiation/commitment during expansion culture (data not shown).

## Discussion

In the present study, we demonstrated through *in vitro* and *in vivo* analysis that human SK-Cs could be divided into two typical cell populations that differentiate into: (1) a myogenic lineage, sorted as human Sk-DN/29^+^ cells; and (2) peripheral nerve support and vascular cell lineages sorted as human Sk-34 cells. It is also possible that human Sk-DN cells are mainly composed Pax7^+^ putative satellite cells, which was confirmed by cell behaviors (tube formation) *in vitro*, and *in vivo* muscle fiber regeneration. In contrast, human Sk-34 cells were non-myogenic and supported peripheral nerve and blood vessel tissue reconstitution. Consequently, human skeletal muscle contains stem or progenitor cells that are able to differentiate into muscle-nerve-blood vessel unit-related cell lineages. This corresponds to the results of our previous reports in mouse skeletal muscle (Tamaki et al., 2005, 2007a,b). However, mouse Sk-DN cells are situated hierarchically upstream of Sk-34 cells (Tamaki et al., 2008b), and exert the same cell differentiation and tissue reconstitution capacities for damaged muscle-nerve-blood vessel units (Tamaki et al., 2007a,b). At present, the relationship between human Sk-DN/29^+^ and Sk-34 cells is unknown. However, when these two cell populations are cultured/expanded together, differentiation/commitment of both cells was unnecessarily accelerated, and *in vivo* cell engraftment efficiency and tissue reconstitution capacity was markedly reduced. This may be profoundly detrimental to the subsequent ability to regenerate skeletal muscle *in vivo* (Montarras et al., 2005). Thus, Sk-DN/29^+^ and Sk-34 cells should be expanded separately.

**Figure 10:**
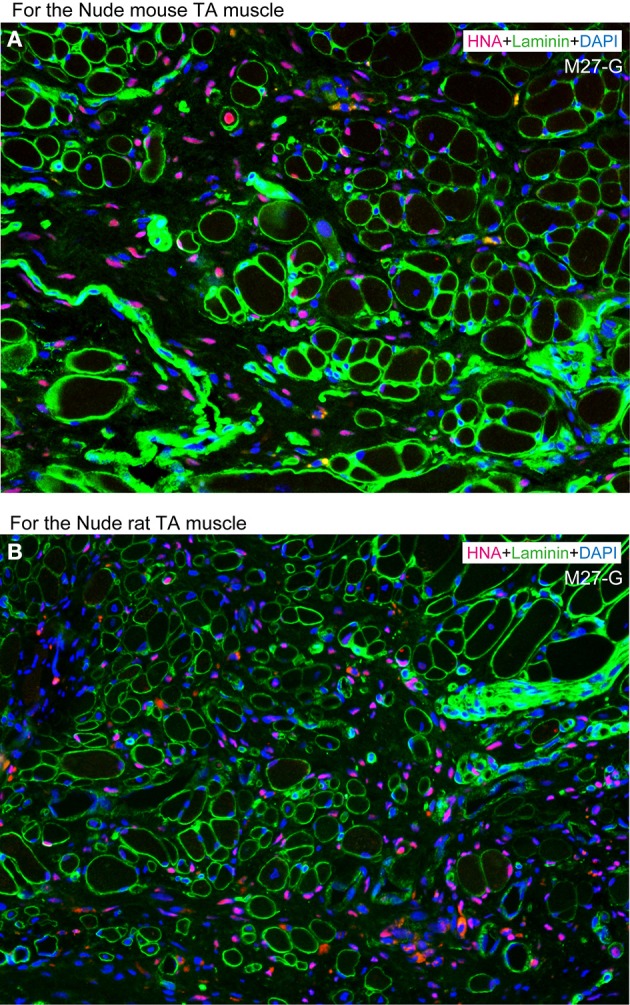
**Typical engraftment of human Sk-DN/29^+^ and Sk-34 cells at 8 weeks after co-transplantation. (A)** Co-transplantation into severely damaged tibialis anterior (TA) muscle of nude mouse. **(B)** Co-transplantation into severely damaged tibialis anterior (TA) muscle of nude rat. Favorable and active engraftment of HNA^+^ nuclei into muscle fibers and interstitium was observed under both transplantation conditions. Therefore, synergistic effects are promising in co-transplantation of Sk-DN/29^+^ and Sk-34 cells, while co-expansion culture showed poor results. This is similar to the case of solo transplantation of mouse Sk-DN or Sk-34 cells (see References. Tamaki et al., 2005, 2007a,b). In comparison to solo human Sk-DN/29^+^ transplantation in Figure 6, the number of fibers including HNA^+^ nuclei is likely low. This may depend a reason that the number of transplanted Sk-DN cells were absolutely reduced half because of the addition of Sk-DN + Sk34 cells, under adjustment to the total cell number of solo transplantation.

However, co-transplantation of separately expanded both cells showed synergistic effects, as there were large number of HNA^+^ cells detected in both the interstitial space and in the muscle fibers (Figure 10). Thus, the combined or separate use for regeneration of myofibers and/or peripheral nerve-blood vessel networks is possible, depending on therapeutic application, and this is considered to be one of the beneficial points of the present method.

Several stem cell populations in the human skeletal muscle have been sorted by FACS in the freshly isolated state (Zheng et al., 2007, 2012; Vauchez et al., 2009; Lecourt et al., 2010; Pisani et al., 2010; Bareja et al., 2014). However, the characteristics of isolated cells do not correspond between previous reports and the present study. The most important point is the inclusion or detection of CD56^+^ cells in the freshly isolated state, and this is common to the above reports, whereas our method did not include CD56^+^ cells. Cells having differentiation potential for myogenic and endothelial cells were fractionated as myoendothelial cells co-expressing CD56, CD34 and CD144, in contrast to other CD56^+^ myogenic cells (Collins et al., 2005; Zheng et al., 2012). Myogenic populations were also sorted as CD56^+^ and CD34^+/−^ cells in relation to their association with adipogenic potential (Pisani et al., 2010), and with additional use of CD15 (Lecourt et al., 2010). On the other hand, myogenic/adipogenic cells were also isolated using aldehyde dehydrogenase activity (ALDH) and CD34^+/−^ reactions (Vauchez et al., 2009), but were negative for CD31, CD45, CD56 and CD140b, which is similar to the present study, but this report actually included 20 min of pre-incubation with Aldefluor assay buffer. Thus, it is also unclear how this treatment affects the expressions of these markers.

Differences in the basic cell isolation method were considered to have a strong effect on this issue (Collins et al., 2005). Particularly, when using enzymatic digestion, proteolytic contamination can damage cell surface ligands or receptors, which are necessary for stem cell function after *in vivo* transplantation (Collins et al., 2005). We have thus consistently used lower concentrations of collagenase (0.1%) in DMEM and always add 5–10% FBS to the collagenase solution in order to minimize contaminating protease activity and to protect isolated cells. This is a milder treatment when compared with previous studies, which have used higher concentrations of collagenase (0.2–2.0%) without suppression of protease activity, followed by dispase (0.25%) and/or trypsin (0.1–0.25%) treatments. Furthermore, we never mince donor muscle before enzymatic digestion in contrast to the all other reports, and sample dissection is kept to a minimum (to produce 6 × 6 × 40 mm samples; see Methods). Therefore, there are several reasons that our isolated cells showed fewer available cell markers and better differentiation potential after transplantation than in other reports. This is also the case in our previous mouse skeletal muscle experiments, in which cell isolation resulted in minimal contamination from other cell types, such as committed endothelial cells. In fact, we first identified Myo-endothelial cells from mouse skeletal muscle as Sk-34 cells, but there were no CD31^+^ cells present among them (Tamaki et al., 2002), nor were there CD133^−^ or NCAM (CD56)-expressing cells (Tamaki et al., 2008a) when using our method. Consequently, the present human cell surface marker expression was similar to our previous mouse study, but did not correspond to other reports of the mouse or human skeletal muscle.

On the other hand, the present results also indicate that cell surface marker expression after culture are not useful for the classification of cellular differentiation capacity, as there were no typical differences observed between Sk-DN and Sk-34 cells after culture (Figure 1A), while the actual cell behaviors in culture (Figures 2–5) and cell differentiation potential *in vivo* (Figures 6–9) were clearly different. Therefore, a direct comparison of the characteristics in the human Sk-Cs with cells fractionated by other researchers after expansion and/or series culture (Young et al., 1999, 2001; Alessandri et al., 2004; Wei et al., 2011; Chirieleison et al., 2012) is difficult. Nonetheless, we believe that long-term culture of Sk-Cs, including satellite cells, has a profoundly detrimental effect on their subsequent ability to regenerate skeletal muscle *in vivo*, because cells begin to differentiate in culture (Montarras et al., 2005).

With regard to therapeutic potential, cellular engraftment capacity and cryopreservation are also important factors. In previous studies, the immunodeficient SCID mouse (Skuk et al., 2006; Zheng et al., 2007, 2012; Meng et al., 2011; Chirieleison et al., 2012) or more specific animals, such as Rag2^−/−^γc^−/−^ mouse (Cooper et al., 2001; Vilquin et al., 2005; Riederer et al., 2008; Pisani et al., 2010; Meng et al., 2011) have generally been selected as recipients animals, in order to obtain better cell engraftment ratios. However, we used the athymic nude mouse, which meets the minimum requirements of a recipient animal model for cell transplantation, and sufficient engagement was observed (see Figures 6–9). The results were not significantly different when nude rats were used as recipients (compare Figures 10A,B). Therefore, this may indicate a higher engraftment capacity for present human Sk-DN and Sk-34 cells. In addition, we also confirmed that cryopreservation is suitable for the present human Sk-Cs. The most effective and practical cryopreservation method is when enzymatically isolated cells are washed with DMEM/10% FCS, immersed in cell preservative solution, set into a specific cell freezing container, and then stored at −80°C. After 24 h, cells can be transferred to liquid nitrogen and stored. Stored cells were thawed and awaked at 37°C, washed with DMEM/10%FCS, and prepared for standard CD marker staining and cell sorting. Using this cryopreservation method, weaker cells are lost, but sufficient numbers are maintained, and the capacities for consecutive cell sorting, expansion culture and *in vivo* engraftment/differentiation remained favorable.

**Figure 11:**
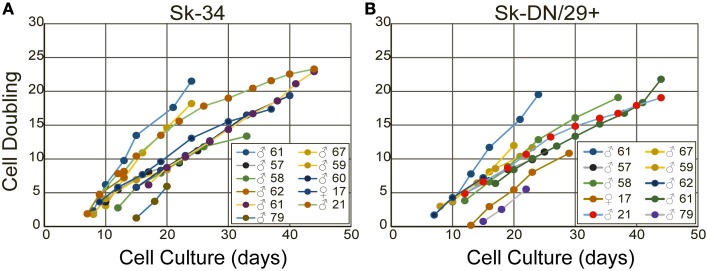
**The cell doubling of Sk-34 (A) and Sk-DN/29^+^ (B) cells**. Note that there are not all samples appeared, because some were directly used for *in vivo* transplantation study, and some were used for immunocytochemical study.

Finally, there were no significant effects of the age, gender and muscle region for the isolated cellular proliferation/differentiation capacities observed between samples, whereas the age of subjects ranged 17–79. Superficially, it is curious, because the age dependent decrease in muscle mass, motor functions, and ability of regeneration is generally occurring as the sarcopenia and/or dynapenia (Clark and Manini, 2008; Aagaard et al., 2010). In addition, which muscles would be preferred for harvesting stem cells for future regeneration therapies are important and interesting. However, present results indicated that there were no significant differences in the proliferation/differentiation/engraftment abilities of resident cells from the variety of muscles with age and gender, when there were isolated and exposed the same condition in culture or transplanted in the damaged muscles of immunodeficient animals. Of course individual differences apparently existed between samples, but there were no constant tendencies in the age, gender and muscle type and regions as shown in Figures 11A,B and Supplemental Figure 3. Therefore, present results further encourage to a notion that *in vivo* cellular environment, such as the cachexia (Thomas, 2007; Cruz-Jentoft et al., 2010; Berardi et al., 2014) with reduced physical activities, may be a major cause for sarcopenia and/or dynapenia in the human skeletal muscle. However, once they were isolated and exposed the same condition, individual differences were reduced. Consequently, the Sk-Cs therapy may be effective to repair muscle regardless of the age, sex or muscle region that is used as a donor for muscle stem cells.

In conclusion, the novelty of the present study is a clear separation of the myogenic and the other multipotent (nerve/vascular) stem cells are possible in the human Sk-Cs, whereas this is difficult in the mouse. In addition, we strongly recommend the following conditions for therapeutic use of human skeletal muscle-derived cells. (1) For expansion culture, dexamethasone should not be used continuously in the proliferation medium, but may be used within the first week in the awaking medium. (2) Cell expansion culture should be limited to 3 passages for 21 days in total. (3) Cell expansion culture should not be perform on enzymatically isolated bulk cells; cells should be divided into Sk-DN/29^+^ and Sk-34 fractions before culture. These three points are important for eliminating the risks of karyotype abnormalities, and will ensure sufficient safety. In addition, the following conditions are necessary to obtain better *in vivo* transplantation results. (1) Muscle samples should not be minced before enzymatic digestion, and a milder enzymatic cocktail should be used as a minimal digestive requirement (inhibit contaminating proteases, etc.). If the residual muscle was apparent in this step, repeated (additional) isolation for 1 h is available after changing to a new enzymatic cocktail, while the cell survival ratio is reduced. (2) Cell expansion culture should be performed separately for Sk-DN and Sk-34 cells, as the cell engraftment ratio and differentiation capacities *in vivo* are significantly reduced in combined culture. (3) In addition, significant reductions of *in vivo* engraftment capacity were also observed beyond 2 passages, even after separate expansion, and this trend is more apparent for the myogenic potential of Sk-DN cells. It is therefore possible that the present method revealed efficient, stable and safety expansion of the human skeletal muscle-derived stem cells, and this also contributed to an active engraftment *in vivo* after transplantation.

### Conflict of interest statement

The authors declare that the research was conducted in the absence of any commercial or financial relationships that could be construed as a potential conflict of interest.

## Supplementary material

Supplemental Figure 1 **Behavior of Sk-DN/29^−^ cells after 62 days culture with 3 passages**. Debris are still observed **(A)**. Cells are typically showing flat and less characteristics **(B,C)**.

Supplemental Figure 2 **Determination of HNA^+^ cells in the immunoelectron microscopy**. The method-1 is a using of higher-brightness view as shown in **(A)**. Black dots densities in the HNA^+^ nuclei (black arrows) are clearly higher than negative nuclei (red arrows). However, this view is not suitable for general morphology. Method-2 is a using of high magnification photograph as shown in **(B)**. In this magnification, DAB products can be detected even in the rare black dots densities (black arrows). Bars in **A** = 10 μm, and in **B** = 2 μm.

Supplemental Figure 3 **Side-by-side comparison of engraftment capacity of the young (17 years-old), middle-aged (45 years-old), and old (79 years-old) subject derived Sk-34 and Sk-DN cells after 6 weeks of transplantation**. Age, gender, and muscle region depend particular trends are not detectable in these photographs. **(A,B)** nude mouse TA muscle, **(C,D)** node rat TA muscle.

## Abbreviations

Sk-Cs: skeletal muscle-derived cells
Sk-DN/29^+^ cells: CD34^−^/CD45^−^/CD29^+^ cells
Sk-34 cells: CD34^+^/CD45^−^
FACS: fluorescence activated cell sorting
DMEM: Dulbecco's modified essential medium
FCS: fetal calf serum
IMDM: Iscove's modified Dulbecco's medium
TA: tibialis anterior
G: gastrocnemius
S: soleus
A: rectus abdominis
VM: vastus medialis
VL: vastus lateralis
PBS: phosphate-buffered saline
PFA: paraformaldehyde
PB: phosphate buffer
SMA: smooth muscle action
DAPI: 4′,6-diamino-2-phenylindole
DAB: 3,3′-Diaminobenzidine
SkMA: skeletal muscle actin
HNA: human nuclear antigen.
