# Supplementary figures and images for: Therapeutic isolation and expansion of human skeletal muscle-derived stem cells for the use of muscle-nerve-blood vessel reconstitution
Source: Front Physiol. 2015 Jun 2;6:165. doi: 10.3389/fphys.2015.00165 (PMC4451695; doi:10.3389/fphys.2015.00165)

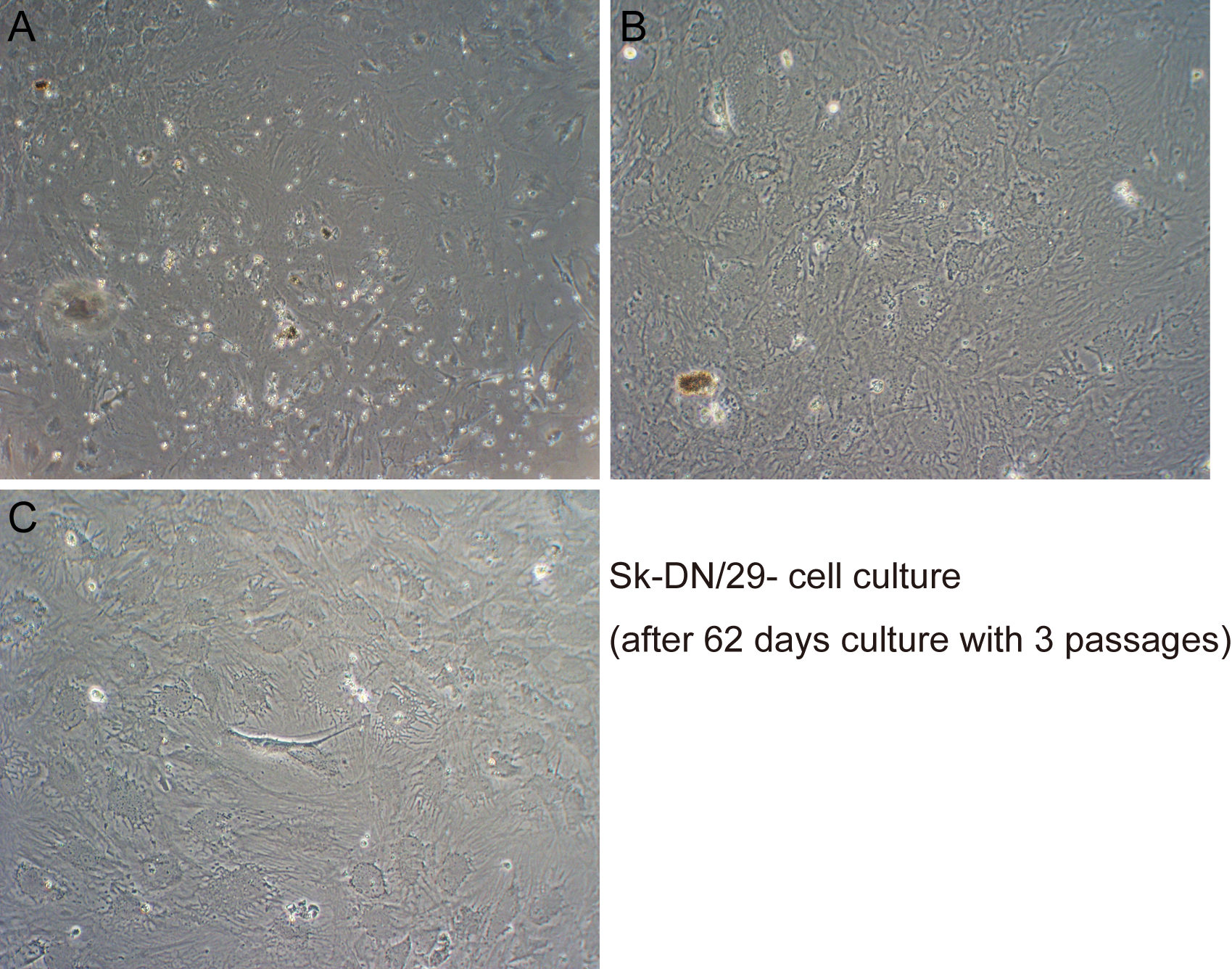

Supplement: Supplemental Figure 1 — Behavior of Sk-DN/29− cells after 62 days culture with 3 passages. Debris are still observed (A). Cells are typically showing flat and less characteristics (B,C). [file Image1.TIF]

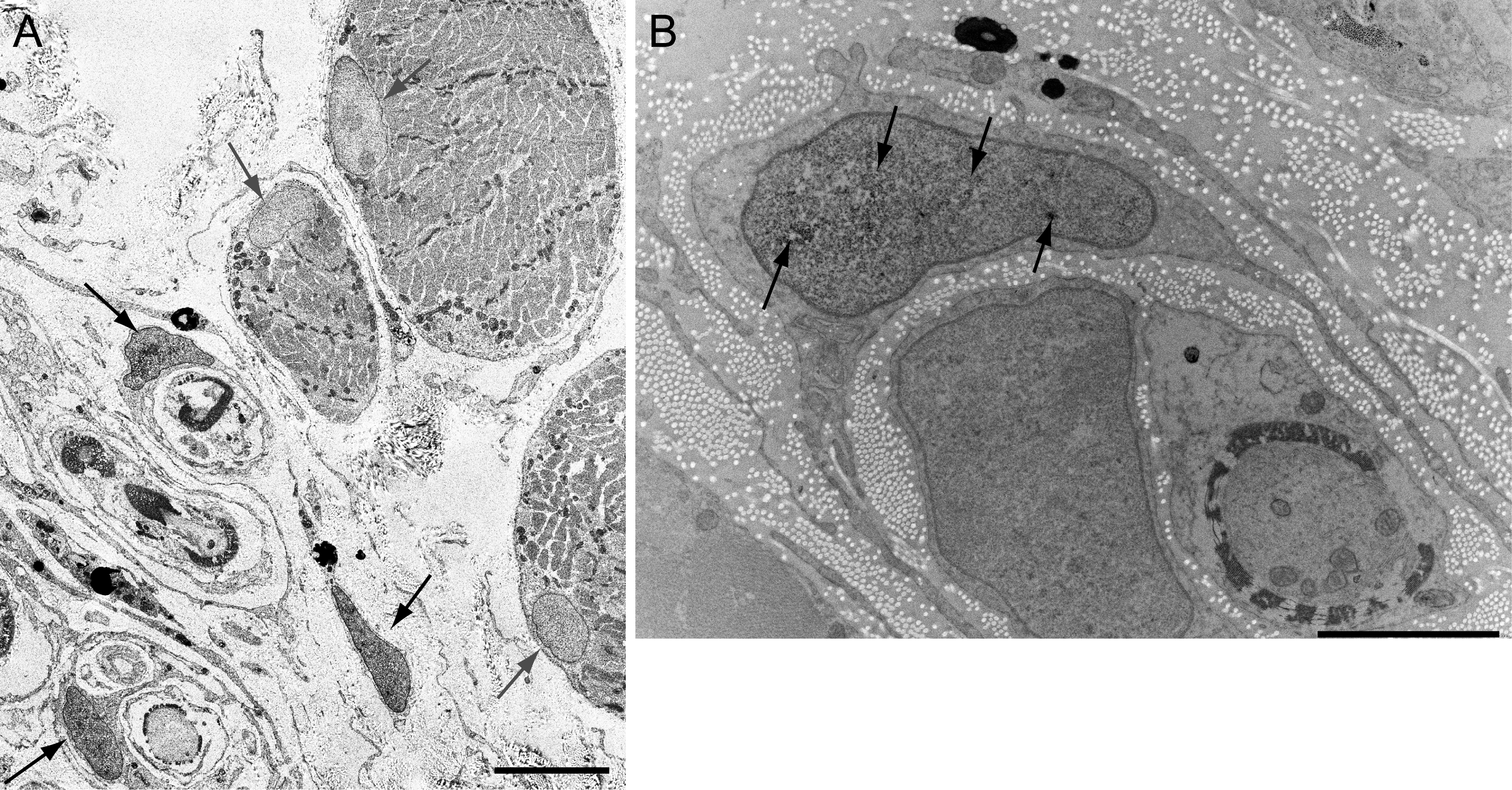

Supplement: Supplemental Figure 2 — Determination of HNA+ cells in the immunoelectron microscopy. The method-1 is a using of higher-brightness view as shown in (A). Black dots densities in the HNA+ nuclei (black arrows) are clearly higher than negative nuclei (red arrows). However, this view is not suitable for general morphology. Method-2 is a using of high magnification photograph as shown in (B). In this magnification, DAB products can be detected even in the rare black dots densities (black arrows). Bars in A = 10 μm, and in B = 2 μm. [file Image2.TIF]

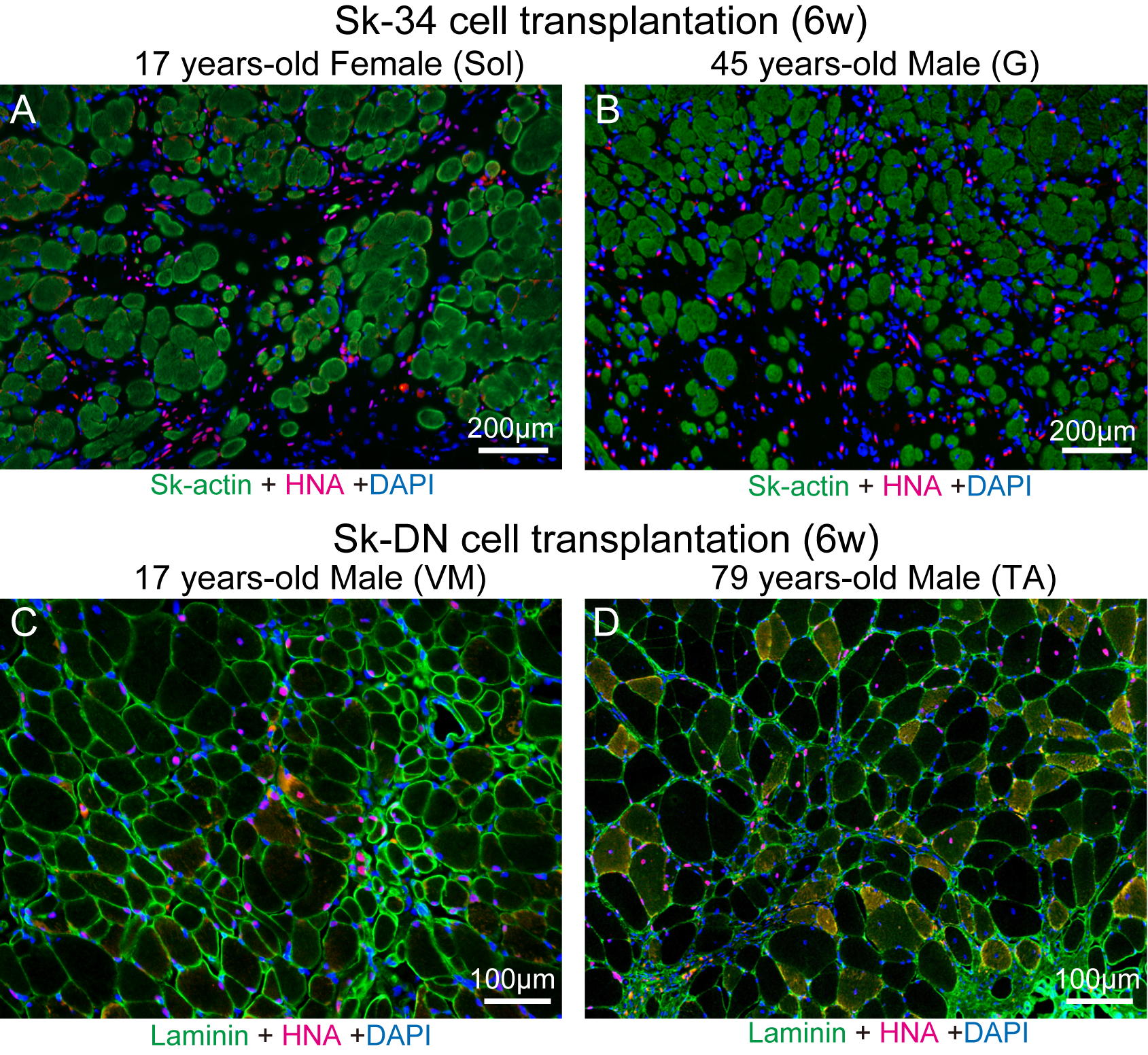

Supplement: Supplemental Figure 3 — Side-by-side comparison of engraftment capacity of the young (17 years-old), middle-aged (45 years-old), and old (79 years-old) subject derived Sk-34 and Sk-DN cells after 6 weeks of transplantation. Age, gender, and muscle region depend particular trends are not detectable in these photographs. (A,B) nude mouse TA muscle, (C,D) node rat TA muscle. [file Image3.TIF]
