# Supplementary material for: Therapeutic isolation and expansion of human skeletal muscle-derived stem cells for the use of muscle-nerve-blood vessel reconstitution
Source: Front Physiol. 2015 Jun 2;6:165. doi: 10.3389/fphys.2015.00165 (PMC4451695; doi:10.3389/fphys.2015.00165)
Supplement: Supplementary file 4 [file Table1.DOCX]

| Additional Table 1. Analyzed cytokines and other materials for human Sk-34 and Sk-DN cell culture. | | | | | |
| --- | --- | --- | --- | --- | --- |
| No. | Materials | Name of products | Manufactures, City, State or Country | Final concentration |  |
| 1 | HGF | Recombinant Human HGF | HUMANZYME, Chicago, IL | 20ng/ml |  |
| 2 | bFGF | Recombinant Human FGF basic | PEPROTECH, Rocky Hill, NJ | 12.5ng/ml |  |
| 3 | EGF | Recombinant Human EGF | PEPROTECH, Rocky Hill, NJ | 40ng/ml |  |
| 4 | IGF-1 | Recombinant Human IGF-I | PEPROTECH, Rocky Hill, NJ | 20ng/ml |  |
| 5 | Dexamethasone | DECADRON | Banyu, Tokyo, Japan | 4mg/ml |  |
| 6 | β-Mercaptoethonol | 2-Mercaptoethanol | Wako Pure Chemical, Osaka, Japan | 0.1mM |  |
| 7 | Vitamin E | α-Tocopherol | SIGMA Life Science, Tokyo, Japan | 10nM |  |
| 8 | Vitamin C | L-Ascorbic acid | SIGMA Life Science, Tokyo, Japan | 2mg/ml |  |
| 9 | Hyaluronic acid | Artz | Kaken Pharmaceutical, Tokyo, Japan | 3.3mg/ml |  |
| 10 | Anabolic Androgenic Steroid | Nandrolone dcanoate | Organon, Tokyo, Japan | 0.625ng/ml |  |

| Additional Table 2. Specific primers for human cells. | | | |
| --- | --- | --- | --- |
| Gene name | product size (bp) | Forward primer | Reverse primer |
| MyoD | 173 | GTGCACTCCGGTCCCAAATG | CACCACACACCATGCCTCAG |
| Myf5 | 417 | TGAGAGAGCAGGTGGAGAACTAC | GCCTTCTTCTTCCTGTGTATTAG |
| Pax3 | 215 | GCCCGGACATGTCTTGCTAAC | TTGCCAAAGCATCCATGAGG |
| Pax7 | 288 | CGGCGTTCAACCACCTTCTG | CCGGGTTCATGTGGTTGGAG |
| c-met | 181 | CGGGGAAACATCCCATCAAC | TCAGCTGCAGGTATAGGCAGTGAC |
| M-cad | 123 | GTCTGGGGGCAGAACCTGAG | GGAGGCAAAGGTGGGACTAGG |
| Myogenin | 150 | GGGGCCAAACTTTTGCAGTG | AGAGGCCCCAACCCCTTTTC |
| Skeletal muscle actin (Sk-MA) | 597 | CGTGGCTACTCCTTCGTGAC | CCCATTGAGAAGATTCGTCG |
| Scn1b | 209 | CACATTGGCCGCTTCAGACAC | GGGGCAGGAGTGCATTACAGG |
| NCAM | 222 | CATGGCCAAGTTCTGCTGGAG | GCCCCCGAAGTACAGAATGC |
| p75 | 184 | GCACCACCGACAACCTCATC | ATGCCACTGTCGCTGTGGAG |
| Pmp22 | 200 | AACTCCATCTCGCCCCTTCC | TTGGGCATTTTGTCCGTGTG |
| SMA | 100 | TCCTGCTCCTCTCTGTCTCTAGCA | AACGAGTCAGAGCTTTGGCTAGGAA |
| TEK | 112 | GCTTGCTCCTTTCTGGAACTGT | CGCCACCCAGAGGCAAT |
| VEGF | 200 | ACCCATGGCAGAAGGAGGAG | ACTCCAGGCCCTCGTCATTG |
| Laminin-b2 | 95 | GCGTGCTTCAAGCCATCAAC | TGTGGGGCAGTGCTAGGAAC |
